# AI-Generated Fall Data: Assessing LLMs and Diffusion Model for Wearable Fall Detection

**DOI:** 10.3390/s25165144

**Published:** 2025-08-19

**Authors:** Sana Alamgeer, Yasine Souissi, Anne Ngu

**Affiliations:** 1 Department of Computer Science, Texas State University, San Marcos, TX 78666, USA; angu@txstate.edu; 2 College of Computing and Informatics, University of North Carolina, Charlotte, NC 28223, USA; yasinesouissi@gmail.com

**Keywords:** fall detection, large language models, synthetic data generation, text-to-text generation, text-to-motion generation, time-series analysis, diffusion models

## Abstract

Training fall detection systems is challenging due to the scarcity of real-world fall data, particularly from elderly individuals. To address this, we explore the potential of Large Language Models (LLMs) for generating synthetic fall data. This study evaluates text-to-motion (T2M, SATO, and ParCo) and text-to-text models (GPT4o, GPT4, and Gemini) in simulating realistic fall scenarios. We generate synthetic datasets and integrate them with four real-world baseline datasets to assess their impact on fall detection performance using a Long Short-Term Memory (LSTM) model. Additionally, we compare LLM-generated synthetic data with a diffusion-based method to evaluate their alignment with real accelerometer distributions. Results indicate that dataset characteristics significantly influence the effectiveness of synthetic data, with LLM-generated data performing best in low-frequency settings (e.g., 20 Hz) while showing instability in high-frequency datasets (e.g., 200 Hz). While text-to-motion models produce more realistic biomechanical data than text-to-text models, their impact on fall detection varies. Diffusion-based synthetic data demonstrates the closest alignment to real data but does not consistently enhance model performance. An ablation study further confirms that the effectiveness of synthetic data depends on sensor placement and fall representation. These findings provide insights into optimizing synthetic data generation for fall detection models.

## 1. Introduction

According to a report published by the World Population Prospects [1], the number of people aged 65 and older is projected to double by 2050, which will surpass the number of children under the age of 5. Another report [2] published by the World Health Organization (WHO) states that the global proportion of individuals aged 60 and above is expected to increase from 11% to 22%, growing from around 605 million to nearly 2 billion over the coming decades. This demographic shift brings major health concerns for the elderly, with falls being a significant issue. Therefore, it is crucial to develop effective measures to monitor and manage falls.

**Figure 1:**
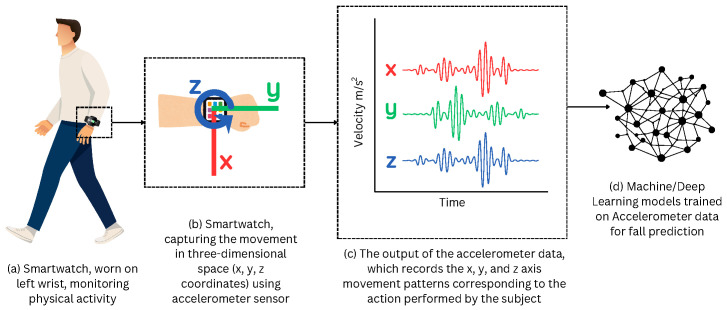
Illustration of smartwatch-based fall detection: (**a**) A person wearing a smartwatch equipped with inertial sensors, which continuously monitor movement. (**b**) Representation of the internal accelerometer of the smartwatch, capturing movement along three axes: *x* (red), *y* (green), and *z* (blue). (**c**) The accelerometer data, corresponding to the *x*, *y*, and *z*-axes, are visualized as time-series signals. (**d**) This data is then processed by machine/deep learning algorithms to detect patterns indicating a fall.

In response to this need, fall detection systems based on wearable sensors, known as wearable sensors-based human activity recognition (WSHAR), have become essential tools in elderly care to prevent serious injuries and enable timely medical intervention. Wearable sensors contain inertial measurement units (IMUs), primarily accelerometers, that generate accelerometer data capturing the wearer’s movements in real-time. This accelerometer data is used by machine/deep learning models for training, and deployed on wearable devices, such as smartphones or smartwatches, to continuously monitor the user’s activity and detect falls as they occur [3]. Figure 1 illustrates the application of smartwatch-based fall detection.

To ensure accurate fall detection and minimal false alarms, a substantial amount of accelerometer data, representing various fall scenarios, must be collected for training the models. However, collecting real fall data from elderly individuals is challenging because it is both impractical and ethically problematic due to the risks involved. Consequently, researchers often make use of fall data generated by younger individuals to train these models. This approach, however, introduces significant discrepancies in model performance, as the movement patterns and accelerometer data produced by young and elderly people may differ. The mismatch in data characteristics often leads to a high rate of false positives, which results in reducing the reliability and effectiveness of fall detection systems when deployed in real-world elderly settings.

To address this challenge, generating synthetic data presents a potential solution to simulate fall scenarios that closely resemble the movements of elderly individuals. One promising approach involves using Large Language Models (LLMs) to generate synthetic data. These models have demonstrated success in other healthcare domains, such as patient monitoring and activity recognition [4,5,6,7,8], by leveraging their advanced reasoning and data generation capabilities. Given the semantics of pre-trained LLMs, it is worth exploring their potential to generate synthetic fall data. To the best of our knowledge, no prior research has systematically investigated the use of LLMs for synthetic fall data generation and its subsequent impact on fall detection systems.

It is important to acknowledge the absence of public domain fall data specifically collected from elderly individuals, which could otherwise be leveraged to fine-tune models for a more accurate representation of real-world fall dynamics in this demographic. Generating age-inclusive fall data is one of our future endeavors. Our focus in this paper is therefore not on generating age-specific movement patterns but on exploring the capability of LLMs to synthesize accelerometer data for generic fall simulations, assessing its impact on a fall detection model trained with a combination of real and synthetic falls, and most importantly, setting the foundation for future advancements in generating elderly-specific fall data. Building on this motivation, this study aims to address three critical research questions:

- Can LLMs generate accelerometer data specific to gender, age, or joint?
- Do LLM-generated data perform better or align more closely with real data than diffusion-based methods?
- Do LLM-generated data improve the performance of the model for fall detection tasks?

By systematically exploring these questions, our major contributions are as follows:

- Data Generation: First, we generate synthetic fall data using two categories of pre-trained LLMs: text-to-motion and text-to-text generation models. These models receive prompts describing fall scenarios and produce accelerometer data accordingly.
- Qualitative and Quantitative Analysis: We conduct both qualitative and quantitative analyses to evaluate the distribution of synthetic data in comparison to real data and assess the degree of alignment between the generated and real data.
- We investigate the impact of augmenting real fall data with synthetic fall data in training a Long Short-Term Memory (LSTM)-based fall detection model. The performance of the augmented model is then compared against a baseline model trained exclusively on real data.
- Ablation Study: Finally, we conduct an ablation study to evaluate the impacts of the quantity of synthetic data, prompting techniques, and baseline dataset characteristics when combined with synthetic data.

The remainder of this document is organized as follows: In Section 2, we discuss the related work, providing an overview of the existing literature on synthetic data generation for fall detection systems. Section 3 details the methodology used in this study, including the synthetic data generation techniques and the machine learning models employed. Section 4 covers the implementation details and experimental setup, including data preprocessing and model evaluation. Section 5 presents the results and discussion, addressing three main research questions. Section 6 provides an ablation study to validate the key findings, and Section 7 summarizes the key findings of this study, where we summarize the insights on the effectiveness of LLM-generated synthetic data in enhancing fall detection model. The document concludes with Section 8, where we present a summary of this work and propose directions for future work.

## 2. Related Work

To position our contribution within both the human activity recognition (HAR) literature and the broader generative AI landscape, we organize prior work into three conceptual families: (i) non-generative data augmentation, (ii) generative simulation-based approaches, and (iii) generative AI with learned representations. Table 1 summarizes these categories, comparing their control over sensor placement, demographic specificity, and temporal fidelity, along with key limitations.

### 2.1. Non-Generative Data Augmentation

Early approaches to address data scarcity in HAR and fall detection relied on traditional oversampling, perturbation, and feature-space transformations. While straightforward to implement, these methods preserve the structure of the source data and thus fail to introduce novel, domain-diverse patterns. For example, Alharbi et al. [9] applied Wasserstein GANs (WGANs) to oversample minority activity classes, reporting F1-score gains of 7–10%. Liaquat et al. [10] evaluated probabilistic autoregressive modeling (SDV-PAR), Time-series GAN (TGAN), and Conditional Tabular GAN (CTGAN), finding that CTGAN-generated data could boost performance but still lagged behind real data accuracy.

**Gap:** These methods improve class balance but lack principled control over domain shift robustness and sensor-specific variability, limiting generalization—a central property emphasized in large-scale generative AI.

### 2.2. Generative Simulation-Based Approaches

Simulation pipelines aim to synthesize sensor data through controllable rendering or biomechanical modeling, and can be categorized as follows. *3D Rendering:* Matthews et al. [11] combined synthetic and real video datasets to train I3D models, improving accuracy by 2%. ElderSim [12] simulated elder movements in customizable environments, improving recognition performance but at high computational cost. *Pose Estimation from Video:* Extracting kinematics from 2D/3D pose estimators [13,14,15] allows indirect generation of time-series data. However, accuracy is sensitive to occlusion, lighting, and motion complexity [16]. *Digital Twins:* OpenSim-based simulations [17] enable biomechanically faithful inertial data generation, offering fine control over movement parameters. Yet, calibration requirements and demographic generalization remain bottlenecks.

**Gap:** While simulation approaches offer higher controllability than GAN-based methods, they often trade off scalability for realism and rarely address adaptability to heterogeneous sensor setups—again, which remains a challenge in generative AI tasks.

### 2.3. Generative AI with Learned Representations

Recent advances in generative AI for motion synthesis leverage representation learning to capture both temporal dynamics and semantic conditioning, categorized into the following types. *Text-to-Motion:* Leng et al. [18] integrated GPT-4 with T2M-GPT [19] to generate virtual IMU data from textual descriptions, improving classifier performance but lacking demographic and sensor-specific control. Meng et al. [20] and Chi et al. [21] incorporated diffusion models for multi-motion synthesis, enhancing coherence but facing diversity–fidelity trade-offs. Wu et al. [22] unified motion captioning and text-to-motion generation but required high-quality training data to maintain biomechanical plausibility. *Cross-domain Generative Uses:* Tang et al. [23] demonstrated LLM-based synthetic data generation for clinical NLP tasks, showing domain transfer potential but without addressing the spatio-temporal constraints of sensor data.

**Gap:** These methods embody the scalability and adaptability central to LLM/diffusion research, yet existing HAR applications do not fully exploit these properties to ensure robust, domain-transferable temporal features.

### 2.4. Observed Challenges and Research Framing

Across all categories, we observe three persistent limitations: (i) limited control over demographic and sensor-specific factors, (ii) weak robustness under domain shifts, and (iii) insufficient application of generative AI principles to enable scalable, cross-domain generalization. Our experimental study systematically evaluates text-to-motion and text-to-text generative models under biomechanical constraints, highlighting both their potential to approximate physically plausible fall patterns and their limitations in achieving robustness across diverse deployment conditions.

## 3. Methodology

In this section, we present a comprehensive overview of the methodology, designed with three guiding principles that are scalability, adaptability, and generalization, to align with best practices in modern generative AI research. At a high level, our pipeline consists of the following: (i) synthetic data generation, encompassing both text-to-motion and text-to-text methods (Section 3.1); (ii) preprocessing steps that preserve dataset variability in sensor placement, sampling rate, and activity distribution while structuring the data for model training and testing (Section 3.2); and (iii) a controlled fall detection classifier (Section 3.3) that isolates the impact of synthetic data on performance.

### 3.1. Synthetic Data Generation

**Figure 2:**
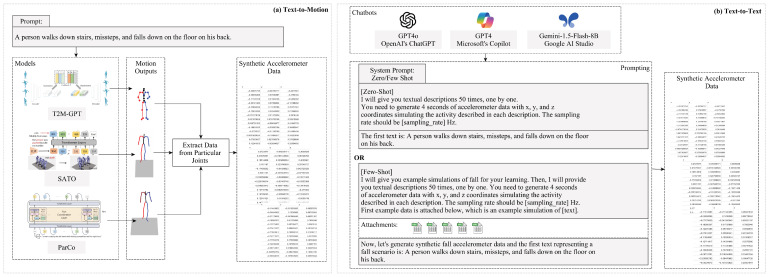
Overview of the data generation process using two categories of pre-trained Large Language Models (LLMs). (**a**) Text-to-Motion: Motion data is generated using models, namely T2M-GPT, SATO, and ParCo, and then, joint-specific data is extracted to create synthetic accelerometer data. (**b**) Text-to-Text: Three LLMs, GPT4o (ChatGPT by OpenAI, USA), GPT4 (Copilot by Google, USA), and Gemini-1.5-Flash-8B (Google AI Studio, USA), take prompts to simulate fall scenarios and generate accelerometer data directly for the left wrist.

This section discusses the generation of synthetic accelerometer data using two categories of pre-trained Large Language Models (LLMs). As shown in Figure 2a,b, the first category is “text-to-motion”, which involves using prompts representing fall scenarios to generate motion data. Then, from this motion data, we extract joint-specific data (including left/right wrist, waist/pelvic). The second category, “text-to-text generation”, consists of using system prompts to set directions, followed by generating accelerometer data with *x*, *y*, and *z* coordinates using the same prompts that reflect fall scenarios. The detailed process for each approach is provided below.

#### 3.1.1. Text-to-Motion Generation

This category involves generating synthetic motion data using pre-trained LLMs that are specifically designed for motion synthesis. We initially constructed a list of 50 prompts, refined from over 200 iterative attempts. Prompts were evaluated by visualizing the generated animations and selecting those that produced biomechanically realistic fall motions. Effective examples included the following: “An elderly person falls down on his right side and lies on the ground” and “A person walks, slips suddenly, and falls on his back on the floor”. In contrast, vague or structurally confusing prompts (e.g., “person moves quickly”) were discarded due to unnatural or static results. Then, we feed these prompts one by one into three motion generation models selected based on their relevance to human motion generation, recent advancements in the field, and ease of use. These models are as follows: Generating Human Motion from Textual Descriptions with Discrete Representations (T2M-GPT) [19], Stable Text-to-Motion Framework (SATO) [24], and Part-Coordinating Text-to-Motion Synthesis (ParCo) [25], which take a descriptive text as input and produce motion data involving multiple joints of the human body.

The T2M-GPT (T2M) model leverages the power of Generative Pre-trained Transformer (GPT) [26] architecture to produce human motion based on textual descriptions. The model utilizes a Vector Quantized-Variational Autoencoder (VQ-VAE) [27] to quantize the motion data into discrete codes, which are then decoded into motion sequences. The ParCo model introduces an innovative approach to text-to-motion generation by focusing on part-based motion generation. Unlike T2M-GPT and SATO, which generate whole-body motions, ParCo decomposes the body into six parts (e.g., right arm, left leg, and backbone) and uses multiple lightweight generators to create motions for each part. These part motions are then coordinated through a Part Coordination module, ensuring that the generated motions are not only fine-grained but also coordinated and realistic. Both T2M and ParCo use transformer-based approaches that focus on the discrete representation of motion. SATO utilizes a diffusion model [28], which is particularly effective in generating high-quality, temporally coherent motion sequences, thereby reducing jitter and instability often observed in other generative models. T2M, ParCo, and SATO are trained on the HumanML3D [29] and KIT-ML [30] datasets, which contain a substantial number of 3D human motion sequences paired with textual descriptions.

These models generate motion data in *.npy* format, from which we extract joint-specific data, located at different indices (Figure 2a), to ensure it aligns with our baseline datasets. Once the relevant data is retrieved, it is stored in a CSV file, formatted with semicolon-separated values, and organized under the headers *x*, *y*, and *z* to represent the respective coordinates.

#### 3.1.2. Text-to-Text Generation

In the text-to-text generation category, we employed three advanced LLMs: GPT4o (OpenAI’s ChatGPT) [31,32], GPT4 (Microsoft’s Copilot) [33], and Gemini-1.5-Flash-8B (Google AI Studio). We chose these LLMs based on their robust capabilities in natural language understanding and generation, as well as their ability to handle complex CSV documents, which was critical for generating few-shot synthetic accelerometer data.

As illustrated in Figure 2b, for generating the accelerometer data, we employed two prompt strategies: zero-shot and few-shot learning. In the zero-shot approach, the LLMs generate data based solely on the provided prompt, without any additional examples. In contrast, the few-shot approach involves supplying the models with examples of the desired output, which improves its ability to produce more accurate and contextually relevant data. Specifically, we used data from five subjects in the baseline datasets in CSV documents, each experiencing different types of falls (back, front, left, right, and rotate), as shots in the few-shot approach. The system prompts for both strategies are shown in Figure 2b.

### 3.2. Baseline Preparation

#### 3.2.1. Baseline Datasets

In this study, we utilized four datasets as baselines: SmartFallMM, KFall, UMAFall, and SisFall. The details of these datasets are summarized in Table 2.

The SmartFallMM (SMM) dataset (Dataset can be downloaded from here: https://anonymous.4open.science/r/smartfallmm-4588 accessed on 8 July 2025) includes data from 16 young (11 male and 5 female, with an average age of 23) and 26 old (12 males and 14 females, with an average age of 65.5) participants. Only young participants were instructed to simulate 5 types of falls (front, back, left, right, and rotational) on an air mattress, in addition to performing 9 types of Activities of Daily Living (ADLs) performed by both young and old participants, with each activity repeated five times. The accelerometer data of falls and ADLs were collected using four types of sensors at a frequency of 32 Hz: Meta Sensors (from MBIENTLAB) on the right wrist and left hip, a Huawei Smartwatch on the left wrist, and a Nexus smartphone on the right hip. For this study, we used accelerometer data collected from the Huawei Smartwatch, worn on the left wrist, and the Nexus smartphone placed on the right hip as baselines. It is important to note that only accelerometer data from young participants were used in all analyses and experiments throughout this study. Data from older participants were referenced solely for comparative analysis when evaluating synthetic samples generated using age-specific prompts (e.g., containing the word ‘elderly’).

The KFall [34] dataset includes simulations from 32 young participants (all males with an average age of 24.9) who performed 21 daily activities and 15 simulated falls, with each activity repeated 5 times. The dataset includes 21 types of ADLs and 15 types of simulated falls (from walking, sitting, fainting, and trying to get up or sit down). Data collection utilized a custom-designed system that synchronizes sensor data with high-frequency video. A nine-axis inertial sensor (LPMS-B2) was attached to the lower back of participants, recording data from a three-axis accelerometer, gyroscope, and magnetometer at a frequency of 100 Hz. For this study, only the accelerometer data was used.

The UMAFall [35] dataset comprises data collected from 17 young participants (10 males and 7 females aged between 19 and 28 years), with sensors placed at five body locations: ankle, waist, right wrist, chest, and right trouser pocket. The dataset includes 8 types of ADLs and 3 types of falls (backward, forward, and lateral falls). In this study, we used accelerometer data of the waist and right wrist only, collected from SimpleLink SensorTag units, at a sampling rate of 20 Hz.

The SisFall [36] dataset comprises data from 38 participants, divided into two groups: 23 young adults (11 males and 12 females aged 19–30 years) and 15 elderly individuals (8 males and 7 females aged 60–75 years). The dataset includes 19 types of ADLs and 15 types of falls (falls from walking, fainting, jogging, and trying to get up or sit down. Sensors were placed at the waist of participants and secured using a belt buckle. Data collection was performed using a self-developed embedded device, which included a Kinets MKL25Z128VLK4 microcontroller, an Analog Devices ADXL345 accelerometer, a Freescale MMA8451Q accelerometer, and an ITG3200 gyroscope. In this study, we used data captured from the ADXL345 accelerometer, at a sampling rate of 200 Hz.

#### 3.2.2. Diffusion-Generated Dataset

To compare the performance of synthetic data generated from text-to-motion and text-to-text models with diffusion-generated data, we utilize Diffusion-TS [37], a cutting-edge generative model specifically designed for time-series data. Diffusion-TS has been extensively validated on various time-series datasets, demonstrating superior data similarity, temporal consistency, and adaptability across different domains. For this study, it serves as a robust benchmark for evaluating the quality and impact of synthetic data in fall detection tasks. We trained the Diffusion-TS model for each dataset and generated 2001 samples, giving us 4 sets of diffusion-generated data for SMM, KFall, UMAFall, and SisFall.

#### 3.2.3. Data Preparation for Training and Testing a Fall Detection Model

To establish baselines, we selected only 12 subjects from all datasets, considering the computationally limited resources and consistency. For training a fall detection model, we grouped the documents (all activities including falls and ADLs of particular subjects) based on subjects using a Leave-Two-Out cross-validation strategy. Specifically, from 12 subjects, data from 8 subjects were used for training, data from 2 subjects were used for validation during training, and data from the remaining 2 subjects were reserved for the final evaluation of the model. This method ensures that the performance of the model is assessed on completely unseen data, which provides a robust evaluation of its generalizability. Then, in each group, we processed the documents to generate the sliding windows, labeling the windows from ADL documents as 0 and those from fall documents as 1. We did not apply any normalization across datasets, as our goal was to preserve inherent differences (e.g., sensor placement; sampling rate) to evaluate how such variability influences the effectiveness of synthetic data in downstream tasks.

To generate synthetic data from Diffusion-TS, we trained it using only fall data of 12 subjects, individually from each baseline dataset, keeping 80% for training and 20% for testing the generated data. For training and testing the fall classifier, we incorporated synthetic data into the training set of each baseline dataset. For instance, merging the SMM dataset with T2M-generated synthetic data created one augmented dataset. Similarly, combining SMM with other text-to-motion and text-to-text models resulted in a total of thirteen distinct augmented datasets. We followed a data-distribution ratio of 60% ADLs, 20% real falls, and 20% synthetic falls.

To evaluate the classifier, we trained the LSTM model separately for each combination of real and synthetic datasets. For example, for SMM, we trained the model using synthetic data from Diffusion-TS, three text-to-motion models, and nine text-to-text models, each added individually to the baseline dataset. In total, we trained 13 models for SMM, excluding the model trained solely on the baseline dataset. The same approach was applied to the other three baseline datasets.

To create sliding windows, we used an overlapping-window technique with a fixed window size of W=128, as our previous work demonstrated its effectiveness in optimizing accuracy [38]. The stride, or step size, was set to 10, meaning that each new window introduced 10 new data points while overlapping with the previous window by 118 data points. This way, we obtained samples of size 128×3, where each of the 128 data points consisted of three coordinates representing the *x*, *y*, and *z* axes of the accelerometer data.

The input samples from each dataset can be represented as follows: Consider an input sequence $S_{i}\in R^{W\times 3}$, where $S_{i}$ denotes the input sample from the *i*-th dataset (out of 4 baseline and the 13 synthetic datasets), and each row $\left(x_{j},y_{j},z_{j}\right)$ corresponds to the *x*, *y*, and *z* coordinates of the accelerometer at time step *j*, with j=1,2,…,W:

(1) $$S_{i}=\left\{\left(x_{j},y_{j},z_{j}\right)|j=1,2,\ldots ,W\right\}$$

where $S_{i}$ represents the input sample from the *i*-th dataset, *W* denotes the number of time steps which is set to 128, and $\left(x_{j},y_{j},z_{j}\right)$ are the accelerometer coordinates at each time step *j*.

### 3.3. Fall Detection Classifier

To train/test a fall detection classifier, and assess the impact of synthetic data on fall detection performance, we use the Long Short-Term Memory (LSTM)-based neural network [39]. LSTM is designed to capture temporal dependencies in sequential data, making it well-suited for analyzing time series such as accelerometer data [38,40,41,42,43]. The model begins with an LSTM layer that consists of 128 units. This layer is followed by a Dense layer with 128 units and Rectified Linear Unit (ReLU) activation [44]. A BatchNormalization (BN) layer is applied to normalize the output. The final Dense layer, with a sigmoid activation function, outputs the prediction. Mathematically, the prediction $\hat{y}$ of model can be described as follows:

(2) $$\hat{y}=\sigma \left(w_{2}\cdot \mathrm{BN}\left(\mathrm{ReLU}\left(w_{1}\cdot \mathrm{LSTM}\left(S\right)+b_{1}\right)\right)+b_{2}\right)$$

where σ is the sigmoid function, $w_{1}$ and $w_{2}$ are weight matrices, $b_{1}$ and $b_{2}$ are bias terms, BN represents batch normalization, and *S* represents the input sample prepared in previous step.

Since model architectures inherently influence fall detection performance, one might assume that selecting a more advanced model could improve results. However, our objective is not to optimize classification performance through model selection but to isolate the effect of synthetic data. We intentionally choose LSTM, a model that does not inherently achieve the highest accuracy to ensure that any observed performance changes are driven by the quality and alignment of synthetic data rather than architectural advantages or parameter tuning. This allows for a more controlled and unbiased evaluation of synthetic data integration.

## 4. Implementation Details and Evaluation Criteria

For training the LSTM model, we standardized the input datasets by removing the mean (to maintain numerical stability in activations) and scaling to unit variance to ensure consistent gradient behavior and improve the training efficiency. We developed the model using the TensorFlow framework, compiled it with binary cross-entropy loss, and optimized it with the Adam optimizer. Early stopping was employed to monitor the training process, with a patience of 50 epochs and a maximum of 250 epochs. Shuffling was set to *True* to introduce randomness in the training process, preventing the model from learning unintended patterns based on data order. A learning rate of 0.001 was consistently applied across all models. The training was conducted on a Dell Precision 7820 Tower, equipped with 256 GB of RAM and a GeForce GTX 1080 GPU.

To ensure robustness and mitigate overfitting, we conducted five iterations of training and testing, where both the subject selection and synthetic data sampling were randomized in each iteration. Specifically, in each iteration, we randomly selected 8 subjects for training, 2 for validation, and 2 for testing. Additionally, 20% of synthetic samples were randomly chosen and incorporated into the training set.

In this study, our primary focus is on the positive class labeled as 1, which represents falls. Therefore, we used the F1-score as the primary metric for evaluating and comparing the performance of the model, as it effectively measures the balance between precision and recall, particularly in the context of imbalanced class distributions. The F1-score is defined as the harmonic mean of precision and recall:

(3) $$\mathrm{F1-score}=2\times \frac{\mathrm{Precision}\times \mathrm{Recall}}{\mathrm{Precision}+\mathrm{Recall}}$$

where Precision is the ratio of true positive predictions to the total predicted positives, and Recall is the ratio of true positive predictions to the total actual positives. The F1-score ranges from 0 to 1, a higher F1-score (closer to 1) for class 1 indicates better model performance in detecting falls, achieving a strong balance between precision and recall, while a lower F1-score (closer to 0) suggests that the model struggles either with false positives (low precision) or missed detections (low recall).

## 5. Results and Discussion

### 5.1. Can LLMs Generate Accelerometer Data Specific to Gender, Age, and Joint?

To determine whether LLMs can generate accelerometer data specific to demographic and sensor placement characteristics, we conducted an experiment comparing neutral and specific prompt conditions. First, we created a set of neutral prompts that described generic fall scenarios without specifying demographic attributes or sensor locations. These prompts avoided references to gender, age, and body joints. We then designed additional prompt groups that explicitly incorporated these characteristics: gender-specific prompts (differentiating between men and women), age-specific prompts (distinguishing between young and elderly subjects), and joint-specific prompts (assigning sensor placements such as the left wrist, right wrist, and waist). In total, we obtained 50×7=350 prompts after permutations.

**Figure 3:**
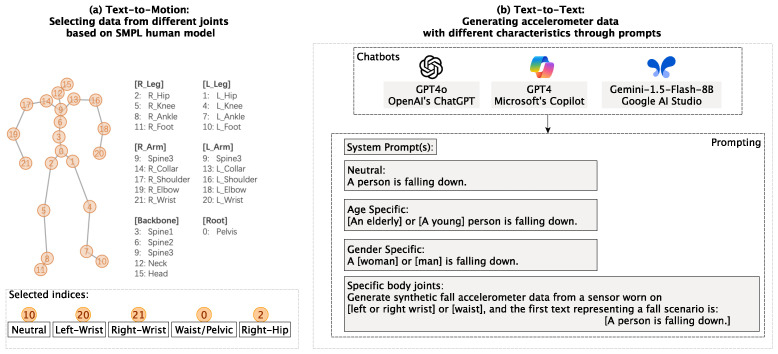
Process for generating synthetic accelerometer data with different characteristics using text-to-motion and text-to-text LLMs. (**a**) Text-to-Motion: Data selection from specific joints based on the SMPL human model, highlighting selected indices for neutral, left wrist, right wrist, and waist/pelvic data. (**b**) Text-to-Text: Prompt-based fall data generation using GPT4o, GPT4, and Gemini (1.5-Flash-8B).

To extract joint-specific accelerometer data from text-to-motion LLMs, we utilized a 22-joint body system based on the widely used Skinned Multi-Person Linear (SMPL) model [45]. As illustrated in Figure 3a, each joint in the SMPL system is represented by a unique index, allowing precise selection of accelerometer data corresponding to specific body locations. Specifically, we selected the relevant indices as follows: the left wrist data was extracted from index 20, the right wrist from index 21, and the waist/pelvic region from index 0. For the neutral case, we deliberately chose the left foot, extracted from index 10. This selection was made to ensure that the reference data was biomechanically distinct from the upper-body sensor placements while still capturing meaningful movement patterns. The foot serves as a valid neutral baseline for two key reasons. First, during falls, lower extremity movements are often less affected by arm-specific actions such as reaching or bracing, making foot data less likely to encode gender- or age-specific motion variations. Second, the left foot provides a stable reference point that does not introduce bias toward common fall recovery mechanisms, which often involve arm or torso movements, thereby minimizing the risk of subtle demographic influences affecting our comparative analysis.

We computed joint-specific accelerometer data from the extracted 3D joint coordinates by calculating discrete second-order differences across consecutive frames. For example, considering the left wrist joint, let p(f)=[x(f),y(f),z(f)] denote 3D positional coordinates at frame *f*. We compute the acceleration vector a(f) as the discrete second derivative of position with respect to time as follows:

(4) $$a\left(f\right)=\frac{p(f+1)-p(f)}{\Delta t^{2}}$$

where Δt represents the time interval between frames (Δt=1/46s). We independently computed each component of acceleration, namely $a_{x}\left(f\right)$, $a_{y}\left(f\right)$, and $a_{z}\left(f\right)$, from positional differences along their corresponding axes, scaling appropriately to obtain units in meters per second squared (${m/s}^{2}$).

For text-to-text models, we generated synthetic accelerometer data by directly prompting the models with different specificity levels. This process is illustrated in Figure 3b. The LLMs were instructed to generate biomechanical time-series outputs based on zero-shot prompts that either included demographic details (e.g., age and gender) or specified joint locations.

After collecting the synthetic accelerometer data from the models of both groups, we employed the Kolmogorov–Smirnov (KS) test [46], a non-parametric method for comparing the distribution of two independent datasets. This test calculates KS statistics and corresponding *p*-values, quantifying the likelihood that the two datasets originate from the same distribution. A *p*-value closer to 1 (above 0.05) suggests that the two datasets are statistically similar, indicating that the LLM did not significantly alter its generated accelerometer data based on the prompt variations. Conversely, a *p*-value close to 0 (below 0.05) suggests that the datasets differ significantly, implying that the LLM incorporated the specified demographic or joint-specific characteristics in a meaningful way.

**Figure 4:**
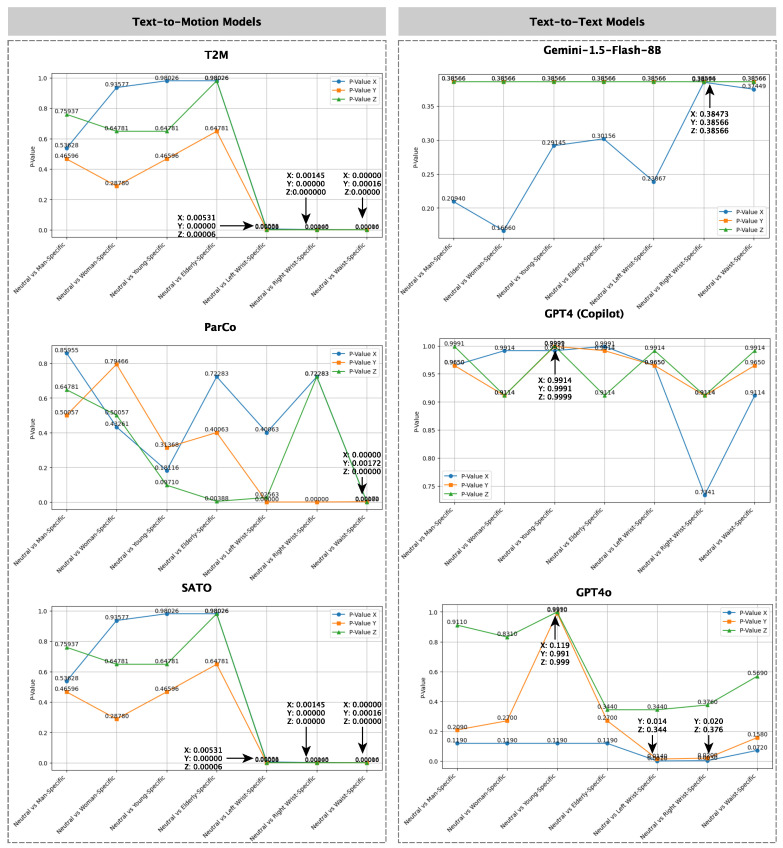
*p*-values from Kolmogorov–Smirnov tests comparing neutral and specific datasets for text-to-motion (T2M, ParCo, and SATO) and text-to-text LLMs (Gemini, GPT4, and GPT40).

The KS test results for text-to-motion models (T2M, ParCo, and SATO) indicate that gender- and age-specific conditions do not produce statistically significant differences from neutral data. As shown in Figure 4 (top row), average *p*-values for Neutral vs Man, Woman, Young, and Elderly comparisons remain high: T2M and SATO range from 0.64781 to 0.98026, while ParCo ranges from 0.19731 to 0.66931, all above the 0.05 threshold. In contrast, joint-specific comparisons show significantly lower *p*-values. For the left-wrist data, T2M and SATO yield 0.00179, and ParCo 0.14209. For the right wrist, values are 0.00081 (T2M and SATO) and 0.48189 (ParCo), while for the waist, all models produce low values: 0.00005 (T2M and SATO) and 0.00057 (ParCo). These results confirm strong statistical differences from neutral data, particularly in the *Y* and *Z* axes (vertical and lateral dynamics). T2M and SATO show consistent distinction across all joints, whereas ParCo displays weaker divergence, especially for the right wrist.

For text-to-text models, average *p*-values for demographic comparisons remain high, 0.685 (GPT4o), 0.733 (GPT4), and 0.707 (Gemini), indicating no significant distributional differences across age or gender. GPT4o shows greater variability in joint-specific prompts, with an average *p*-value of 0.00267, especially for the left and right wrist indices, suggesting moderate sensitivity to sensor placement. In contrast, GPT4 (Copilot) yields consistently high *p*-values (0.733 for demographic; 0.049 for joint-specific), showing limited responsiveness to either condition. Gemini offers intermediate behavior, with joint-specific *p*-values averaging 0.0257, higher than GPT4o for wrist data, implying weaker differentiation. Overall, GPT4o exhibits the highest sensitivity to joint-specific prompts, particularly along the *Y* and *Z* axes. However, the observed distinctions remain less substantial than those produced by text-to-motion models.

**Findings:** The findings demonstrate that text-to-motion models, namely T2M, ParCo, and SATO, are highly effective at capturing joint-specific variations, making them ideal for applications requiring realistic and detailed motion patterns tied to specific sensor placements, such as wearable sensor training for fall detection. This effectiveness likely stems from their training on structured motion datasets, which encode biomechanical constraints across joints. However, these models show limited differentiation for demographic attributes like gender and age, likely due to the absence of demographic conditioning/context in their training data or model architecture. Among text-to-text models, GPT4o emerges as the most responsive, displaying some ability to generate joint-specific variations. These changes, however, are milder than those produced by text-to-motion models, possibly because GPT4o lacks an explicit kinematic structure, making it more suitable for scenarios where moderate variability is sufficient but biomechanical fidelity is not critical.

Based on these findings, we excluded sensor-specific and demographic cues from the prompts to minimize confounding factors. We then curated 50 diverse prompts (Table 3), inspired by incident reports [47], to capture a broad range of fall directions and causes, ensuring both semantic diversity and biomechanical plausibility. These prompts were subsequently used to generate synthetic data, which forms the foundation for the experiments presented in the later sections.

### 5.2. Do LLM-Generated Data Perform Better or Align More Closely with Real Data than Diffusion-Based Methods?

To evaluate the alignment of synthetic fall data with real fall datasets, we conducted two types of analyses: qualitative analysis using visualizations and quantitative analysis using two performance metrics. To prepare comparison pairs for each baseline dataset, we utilized zero-shot (ZS) synthetic data generated by text-to-text models without modification. For example, when comparing baseline SMM data with text-to-text model outputs, we directly used ZS data from each model. In contrast, to generate few-shot (FS) data, we provided each model with examples of falls from five randomly selected subjects per dataset, as prior studies on few-shot have shown that 3–5 examples are typically sufficient for effective conditioning [48]. We made sure that the selected samples captured a representative range of fall directions and body dynamics, enabling robust few-shot generation. For the SMM, KFall, UMAFall, and SisFall datasets, we extracted fall samples from five subjects and submitted them as CSV files, resulting in 12 (4×3) sets of FS accelerometer data across the three text-to-text models. For text-to-motion generation, we extracted data from the relevant joint indices corresponding to each baseline dataset. For instance, for the SMM dataset, we used data from the left wrist (index 20), for KFall and SisFall, the waist (index 0), and for UMAFall, the right wrist (index 21) data (see Figure 3).

For both qualitative and quantitative analysis, we generated 28 synthetic datasets for comparison with four real-world datasets. These included 4 datasets from the Diffusion-TS model, 12 from text-to-text models, and 12 from text-to-motion models.

**Figure 5:**
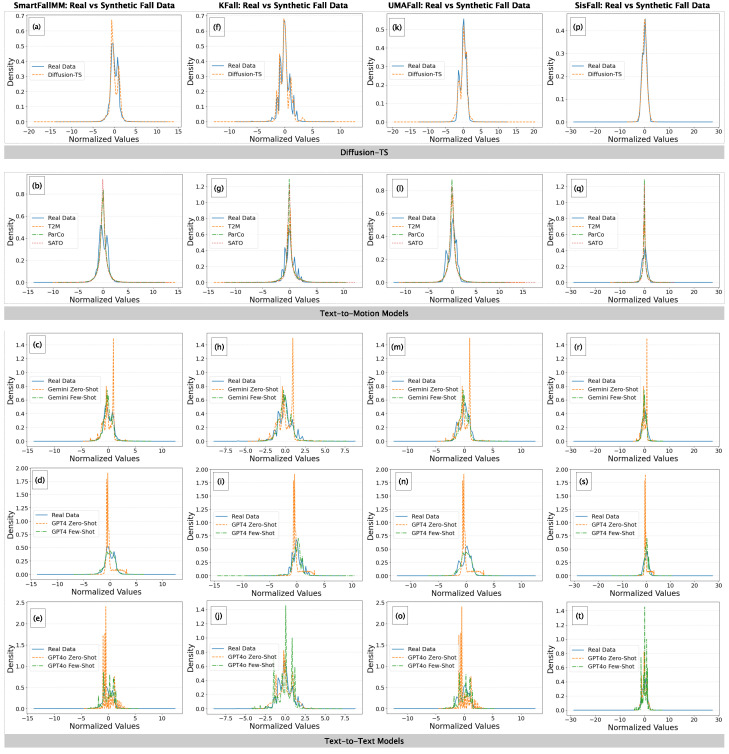
Comparison of normalized value distributions of real falls of four datasets, (**a**–**e**) SmartFallMM, (**f**–**j**) KFall, (**k**–**o**) UMAFall, and (**p**–**t**) SisFall, versus synthetic fall data generated from diffusion-based, text-to-motion, and text-to-text models.

**Qualitative Analysis:** In this analysis, we used line plots to compare the distribution of synthetic falls from diffusion-based, text-to-motion, and text-to-text models with those of real falls from SMM, KFall, UMAFall, and SisFall. As shown in Figure 5, the plots are based on densities computed from the centers of histogram bins, with distinct line styles used for clarity.

*SMM Dataset:* In Figure 5a, Diffusion-TS aligns closely with real data, showing only minor deviations. Among text-to-motion models (Figure 5b), T2M offers the best match, while ParCo and SATO over-concentrate near the mean. In Figure 5c–e, all ZS text-to-text models (Gemini, GPT4, GPT4o) produce sharp central peaks and limited spread. FS prompting improves realism but still shows overestimation near the mean. The results suggest that, for the SMM dataset, text-to-motion models outperform text-to-text models, with Diffusion-TS and T2M providing the closest approximations to real data.

*KFall Dataset:* In Figure 5f, Diffusion-TS aligns well with real data, capturing central tendency and spread. Among text-to-motion models (Figure 5g), T2M matches the real distribution most closely, ParCo shows mild over-concentration near the mean, and SATO produces a pronounced peak, reducing variability. Text-to-text models (Figure 5h–j) show high central peaks in ZS mode, indicating poor generalization. FS prompting improves Gemini and GPT4 alignment by reducing peak intensity. However, GPT4o-FS shows a reverse trend, performing worse than GPT4o-ZS and producing noisier, less representative distributions. Across all models, for the KFall dataset, Diffusion-TS and T2M provide the best alignment with real data. FS prompting generally improves distributional accuracy, but as seen in GPT4o-FS, it can sometimes degrade quality, introducing instability instead of refinement.

*UMAFall Dataset:* In Figure 5k, Diffusion-TS closely tracks real data, preserving both central tendency and variability. Among text-to-motion models (Figure 5l), T2M aligns best, while SATO shows moderate over-concentration near the mean. ParCo produces the sharpest peak, indicating reduced variability and poor generalization. For text-to-text models (Figure 5m–o), ZS variants of Gemini, GPT4, and GPT4o exhibit strong central peaks with limited spread. FS prompting improves variability and alignment for Gemini and GPT4, while GPT4o-FS shows modest improvement but still tends to over-concentrate near the mean. Across all models for the UMAFall dataset, Diffusion-TS and T2M emerge as the most reliable in capturing real data distributions, effectively balancing central tendency and variability.

*SisFall Dataset:* In Figure 5p, Diffusion-TS shows excellent alignment with real data, accurately capturing both central tendency and variability. Among text-to-motion models (Figure 5q), T2M performs best with minimal peak distortion, while SATO and ParCo show increasing over-concentration at the mean. For text-to-text models (Figure 5r–t), ZS variants of Gemini and GPT4 produce sharp central peaks with poor variability, while FS versions improve spread but introduce some noise. Notably, GPT4o-FS (Figure 5t) performs worse than GPT4o-ZS, showing excessive peak height and instability, representing a reverse trend also seen in KFall. Like the other three datasets, Diffusion-TS and T2M demonstrate the strongest alignment with real data for the SisFall dataset.

**Quantitative Analysis:** Our focus is on assessing distribution-level fidelity and variability coverage between real and synthetic datasets, instead of performing pairwise alignment or one-to-one sequence comparisons. Therefore, we employ two metrics for quantitative analysis: Coverage [49] and Jensen–Shannon Divergence (JSD) [50]. Coverage measures the proportion of real data samples represented by synthetic data. Higher values indicate effective capture of real fall variability, suggesting strong generalization. JSD quantifies the statistical similarity between real and synthetic distributions. Lower JSD values signify closer alignment, indicating high fidelity.

**Figure 6:**
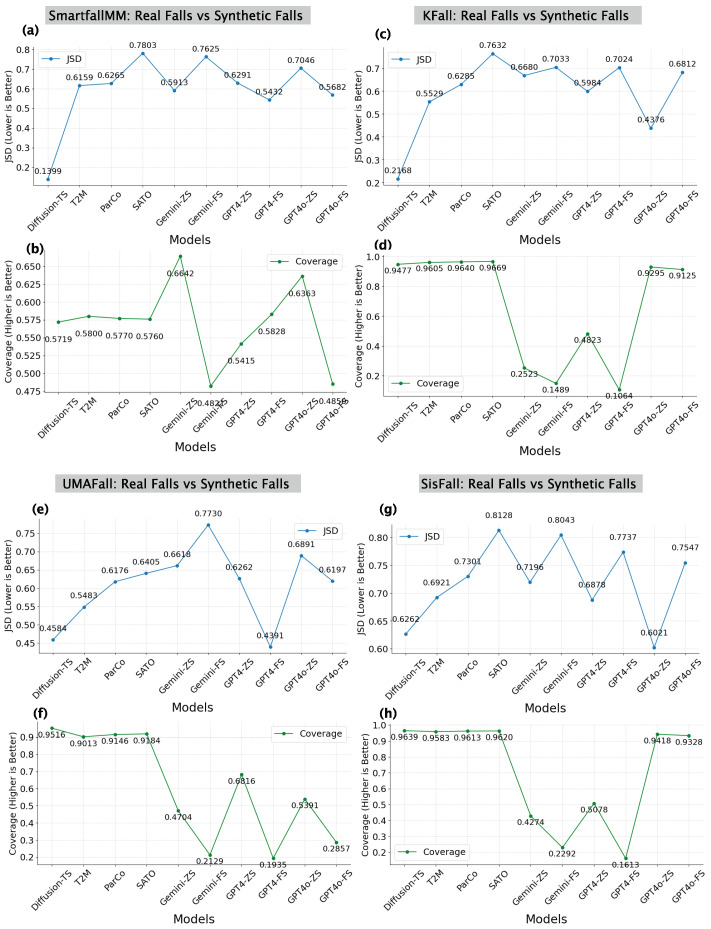
Quantitative comparison of real vs. synthetic falls across four datasets: SmartFallMM (**a**,**b**), KFall (**c**,**d**), UMAFall (**e**,**f**), and SisFall (**g**,**h**), evaluated in terms of Jensen–Shannon Divergence (JSD) and Coverage.

*For the SMM dataset*, Diffusion-TS achieves the best alignment with real data, with the lowest JSD (0.1399) and a moderate Coverage (0.5719), as shown in Figure 6a,b. Text-to-motion models, T2M, ParCo, and SATO, exhibit much higher JSDs (0.6159, 0.6265, and 0.7803), indicating weaker alignment. Their Coverage values (0.576–0.580) are slightly higher than Diffusion-TS but insufficient to offset the divergence. T2M performs best among them, matching its strong qualitative visual alignment. Among text-to-text models, Gemini-ZS achieves the highest Coverage (0.6642) but has a high JSD (0.5913), while Gemini-FS shows the weakest performance (JSD 0.7625; Coverage 0.4821). GPT4-FS yields better alignment (JSD 0.5432) than GPT4-ZS (0.6291), with similar Coverage (0.54–0.58). GPT4o-ZS offers high Coverage (0.6363) but suffers from high JSD (0.7046), while GPT4o-FS improves JSD (0.5682) but drops in Coverage (0.485).

*In the KFall dataset*, Figure 6c,d shows Diffusion-TS with the lowest JSD (0.2168) and highest Coverage (0.9477). T2M, ParCo, and SATO follow with higher JSDs (0.5529, 0.6285, and 0.7632) but Coverage values above 0.96. SATO reaches 0.9669 due to over-concentration near the mean. Gemini-ZS and Gemini-FS perform poorly with low Coverage (0.2523; 0.1489) and high JSDs (0.668; 0.7033). Among GPT models, GPT4o-ZS shows the best alignment (JSD: 0.4376; Coverage: 0.9295), outperforming GPT4-ZS (0.5984; 0.4823) and GPT4-FS (0.7024; 0.1064). However, as observed in Figure 5j, GPT4o-FS exhibited a reverse trend, performing worse than ZS due to increased variance and spikiness. This suggests that the higher JSDs of Gemini-FS, GPT4-FS, and GPT4o-FS may stem from the irregularities and noise introduced.

*For the UMAFall dataset*, Figure 6e,f shows Diffusion-TS with the lowest JSD (0.4584) and highest Coverage (0.9516). Among text-to-motion models, T2M achieves the lowest JSD (0.5483), and SATO the highest Coverage (0.9184), aligning with Figure 5l, where T2M showed the best alignment. Gemini-ZS and Gemini-FS perform poorly with high JSDs (>0.66) and low Coverage (0.4704; 0.2129). GPT4-FS achieves the lowest JSD (0.4391), even beating Diffusion-TS, but its low Coverage (0.1935) indicates limited variability. GPT4-ZS (0.6262 JSD; 0.6816 Coverage) and GPT4o-ZS (0.6891; 0.5391) show better balance. GPT4o-FS performs worse than ZS, with higher JSD (0.6197) and lower Coverage (0.2857) due to added noise and spikiness.

*For the SisFall dataset*, Figure 6g,h shows Diffusion-TS with the lowest JSD (0.6262) and highest Coverage (0.9639). Among text-to-motion models, T2M has the lowest JSD (0.6921) and ParCo the highest Coverage (0.9613), consistent with Figure 5q, where T2M showed better alignment and the high Coverage of SATO resulted from over-concentration near the mean. Gemini-ZS and Gemini-FS perform poorly, with Gemini-FS reaching the lowest Coverage (0.2292) and high JSD (0.8043). GPT4-ZS achieves moderate alignment (JSD: 0.6878; Coverage: 0.5078), while GPT4-FS performs worse (JSD: 0.7737; Coverage: 0.1613). GPT4o-ZS achieves the lowest JSD (0.6021) and high Coverage (0.9418), outperforming GPT4o-FS (0.7547; 0.9328), which suffers from overfitting to few-shot prompts (as observed in Figure 5t).

**Findings:** The qualitative and quantitative analyses indicate that Diffusion-TS consistently outperforms LLM-generated data in aligning with real data across datasets, achieving the lowest JSD and highest Coverage scores. This advantage stems from its direct modeling of statistical patterns in time-series data, leading to better distributional fidelity. In contrast, text-to-text models (e.g., GPT4; GPT4o) show inconsistent performance, with ZS variants often producing sharp peaks and reduced variability due to limited representation diversity. Among LLMs, text-to-motion models like T2M perform better, likely because they are trained on real human pose sequences, enabling them to capture more realistic motion dynamics. However, they still fall short of Diffusion-TS in robustness.

Additionally, FS configurations, particularly GPT4o-FS, degrade performance in high-frequency datasets (KFall: 100 Hz; SisFall: 200 Hz), introducing noise and misalignment. This likely occurs because text-to-text models lack fine-grained temporal control, causing them to oversmooth or inject spurious fluctuations when generating dense time-series data. As the sampling rate increases, these imperfections become more pronounced, amplifying temporal instability. In contrast, Diffusion-TS maintains strong alignment across frequencies due to its stepwise generation process, which preserves temporal consistency and fine detail, highlighting its reliability for generating synthetic fall data.

### 5.3. Do LLM-Generated Data Improve the Performance of the Model for a Fall Detection Task?

To determine whether LLM-generated synthetic data improves fall detection performance, we trained and tested the LSTM model with combinations of real and synthetic fall data. Specifically, we utilized the same 28 synthetic datasets generated in the previous section (see Section 5.2). However, in this analysis, we incorporated the synthetic data into the training sets of the baseline datasets (with 60% of ADLs, 20% of real fall samples, and 20% of synthetic fall samples) and evaluated its impact on model performance. We trained a total of 29 models, including one baseline model trained solely on real data and 28 models trained on a combination of real and synthetic data. We then compared the performance of each model trained with synthetic data against the baseline to assess the effectiveness of data augmentation in improving LSTM performance (see Section 4 for experimental details). Table 4 presents the average F1-scores (for the fall class (class label 1)) of each dataset. The numbers in parentheses represent the percentage change relative to the baseline F1-score.

*SMM Dataset:* Synthetic data reduced model performance across all methods. The largest drop was from GPT4-ZS (−21.89%), while T2M showed the smallest decline (−4.05%), outperforming Diffusion-TS (−8.11%). This aligns with the lower JSD of T2M (0.6159) compared to SATO (0.7803), suggesting better alignment with real data (Figure 6a).

*KFall Dataset:* All methods improved F1-scores. Specifically, T2M achieved the highest gain (+15.94%), consistent with the low JSD of T2M (0.5529). GPT4-FS had the lowest improvement (+5.65%), while GPT4-ZS (+12.87%) and GPT4o-ZS (+15.43%) outperformed their few-shot counterparts. This reveals a reverse trend, where few-shot prompting degraded performance instead of improving it (Figure 6c).

*UMAFall Dataset:* The greatest improvement came from GPT4-FS (+56.83%), with the lowest JSD of all models (0.4391). T2M had the smallest gain (+20.83%) but still exceeded the improvement of Diffusion-TS (+20.30%), indicating generally high utility of synthetic data in this dataset (Figure 6e).

*SisFall Dataset:* GPT4o-ZS achieved the highest gain (+6.83%) and the lowest JSD among all models (0.6021), while GPT4o-FS reduced performance (−7.10%). This confirms the reverse trend where few-shot prompting introduced noise and instability in high-frequency data (Figure 6g).

**Findings:** Our findings show that LLM-generated synthetic data can improve fall detection, but its effectiveness depends on the characteristics of the real dataset and its alignment with the generated data. Particularly in SMM, performance dropped across all methods, primarily due to two factors: (1) the left wrist sensor captured low-variability movements, limiting the usefulness of synthetic data, and (2) a mismatch in data flow, where SMM includes only the impact phase of falls, while synthetic data modeled full transitions, leading to poor alignment.

In contrast, UMAFall exhibited substantial gains. Its low sampling rate (20 Hz) favored text-to-text generation, and its limited fall diversity benefited from the variability introduced by synthetic data. These characteristics enabled better integration and improved model generalization. KFall and SisFall also showed improvement, aligning their transitional fall movements well with synthetic sequences. However, high-frequency sampling (200 Hz in SisFall) introduced instability in text-to-text few-shot (FS) models, particularly GPT4o-FS, which consistently performed worse than its zero-shot (ZS) counterpart—a reverse trend also seen in KFall.

Across all datasets, text-to-motion models (e.g., T2M) more reliably aligned with real distributions than text-to-text models. While FS prompting helped in simpler, low-frequency datasets, it often introduced noise in complex, high-resolution datasets. Diffusion-TS remained stable throughout, but its limited semantic control and variability constrained its impact.

## 6. Ablation Study

To further verify the findings of Section 5, we conducted targeted stress tests to assess the adaptability and stability of LLM-generated synthetic fall data. In these experiments, we altered dataset configurations, prompt sources, and synthetic data proportions to evaluate robustness under domain shifts in sensor placement, sampling rate, and activity diversity. We focused on three key questions:

- Do the baseline dataset characteristics impact the fall detection task using synthetic data?
- Does the quantity of LLM-generated data affect the accuracy of fall prediction models?
- Which prompting strategy is more effective for generating diverse fall data, human-designed scenarios, or self-generated scenarios?

### 6.1. Do the Baseline Dataset Characteristics Impact the Fall Detection Task Using Synthetic Data?

To validate the impact of baseline dataset characteristics on synthetic data effectiveness, we conducted experiments using UMAFall and SMM, modifying their modalities by extracting waist accelerometer data for UMAFall and right hip accelerometer data for SMM. For text-to-motion models, we extracted synthetic data from the right hip (index 2) for SMM and the waist/pelvic joint (index 0) for UMAFall. From text-to-text models, we used the same zero-shot synthetic data, while to generate few-shot data, we incorporated fall samples from five randomly selected subjects. Additionally, we retrained Diffusion-TS with the updated SMM and UMAFall data to generate synthetic samples with the new modalities. After generating synthetic data, we trained and tested the same LSTM model for fall classification using 5-fold cross-validation. The average F1-scores are reported in Table 5.

As shown in Table 5, for SMM (right hip), most synthetic datasets failed to improve performance, with several models leading to a decline in F1-scores. Notably, ParCo (+6.01%) was the only model that achieved a significant improvement, while others such as Diffusion-TS (−3.65%), SATO (−7.62%), and text-to-text models exhibited a performance drop. In particular, Gemini-FS (−15.54%) and GPT4o-FS (−6.63%) showed the most pronounced declines. Notably, the performance drops in SMM are lower than those observed in Table 4, suggesting that modifying the sensor modality to the right hip improved the alignment between synthetic and real data, mitigating some of the negative impact.

Conversely, in UMAFall (waist), synthetic data significantly improved fall detection performance, with multiple models achieving notable gains. Gemini-ZS and Copilot-FS (+27.15%) yielded the highest improvements, followed by Diffusion-TS (+23.92%) and SATO (+19.89%). This suggests that the synthetic data was better aligned with the real data of UMAFALL, likely due to the dataset’s simpler motion structure and fewer fall variations. However, GPT4o Few-Shot (−5.65%) and Gemini Few-Shot (−4.57%) resulted in a decline, indicating potential instability in certain synthetic data generated by text-to-text models.

**Findings:** The results confirm that baseline dataset characteristics significantly influence the effectiveness of synthetic data in fall detection tasks. UMAFall, with its waist-based accelerometer data, lower motion variability, and fewer fall types, benefited from synthetic augmentation, whereas SMM, with right hip data and higher motion complexity, showed limited or negative improvements. These findings suggest that synthetic data is more effective when the baseline dataset has lower complexity and clearer movement patterns but may struggle to align well with datasets that capture more subtle or diverse motion variations.

### 6.2. Which Prompting Strategy Is More Effective for Generating Diverse Fall Data—Human-Designed Scenarios or Self-Generated Scenarios?

The goal of this study is to assess whether human-designed prompts introduce biases or limitations that confuse LLMs, potentially affecting the diversity and realism of synthetic fall data. By comparing the performance of models trained on self-generated vs. human-designed scenarios, we aim to determine which strategy leads to better variability, improved data alignment in zero-shot settings, and ultimately, stronger fall detection performance. For this purpose, we conducted an experiment using text-to-text models exclusively. We did not use text-to-motion models, as they are limited to generating individual motion sequences rather than diverse fall scenarios iteratively. To compare human-designed and self-generated fall scenarios, we formulated the system prompts for LLMs, as shown in Listing 1.

**Listing 1.** Prompting pipeline for generating synthetic fall scenarios and corresponding accelerometer data using an LLM.System Prompt: I want you to create 50 scenarios of persons falling. Each scenario must be unique. List of 50 textual descriptions of fall scenarios. System Prompt: Now, for all of these 50 scenarios, create accelerometer data of 4 s with x, y, z   columns at sampling speed [sampling_rate] Hz. The output CSV must contain 4 columns with names   x, y, and z in lower letters. The CSV must be semicolon-separated. Response: output.CSV

We then used synthetic accelerometer data generated from these prompts to train and test an LSTM model over five folds, with the average F1-scores reported in Table 6.

For SMM, GPT4o-Auto achieved the least performance drop (F1: 0.724, −2.16%), substantially better than GPT4o-ZS (0.588, −20.54%). A similar pattern was observed in GPT4-Auto (0.714) vs. GPT4-ZS (0.578), and Gemini-Auto (0.626) vs. Gemini-ZS (0.636). For KFall, differences between prompting strategies were minor. GPT4o-ZS (0.898, +15.42%) slightly outperformed GPT4o-Auto (0.892, +14.65%). The same trend appeared in GPT4-ZS (0.878) vs. GPT4-Auto (0.874). However, Gemini-Auto (0.854, +9.25%) outperformed Gemini-ZS (0.789, +2.06%). For UMAFall, Auto prompting led to the highest gains. GPT4o-Auto achieved 0.840 (+54.98%) vs. GPT4o-ZS (0.580, +7.01%). GPT4-Auto (0.660, +21.77%) also outperformed GPT4-ZS (0.630, +16.24%), and Gemini-Auto (0.723, +33.39%) outperformed Gemini-ZS (0.575, +6.09%). For SisFall, prompt type had minimal impact. GPT4o-ZS (0.782, +6.83%) performed slightly better than GPT4o-Auto (0.768, +4.92%). Performance was nearly identical between GPT4-Auto (0.774) and GPT4-ZS (0.772), as well as between Gemini-Auto (0.772) and Gemini-ZS (0.752).

**Findings:** The results indicate that auto-generated prompts generally outperform human-designed prompts in generating diverse and realistic fall scenarios, though their impact varies across datasets. For SMM and UMAFall, auto-generated prompts significantly improved performance, demonstrating that LLMs benefit from self-generated variability rather than manually constrained descriptions. For KFall and SisFall, accelerometer data generated from auto-generated prompts showed minimal improvement. This is likely because these datasets already have intrinsic variability that is well aligned with synthetic data, reducing the need for prompt optimization. Similarly, the high sampling rates may have limited the benefits of synthetic data, as LLMs lose specificity regardless of whether prompts are user-designed or auto-generated, highlighting their limitations in high-frequency datasets.

### 6.3. Does the Quantity of LLM-Generated Data Affect the Accuracy of Fall Prediction Models?

To investigate whether increasing the quantity of LLM-generated synthetic data affects the accuracy of fall prediction models, we conducted an LSTM-based experiment over five folds while systematically adjusting the proportion of synthetic data. Specifically, in the training sets, we used 50% ADLs, 10% real falls to retain natural data, and 40% synthetic falls, with the same training protocol.

For text-to-motion models, we combined synthetic data from all three models (T2M, ParCo, and SATO) to meet the required percentage, ensuring that a diverse range of generated motions is incorporated into training, reducing the biases that may arise from any single generative approach. For text-to-text models, we selected the best-performing prompting strategy (ZS or FS) from the previous fall prediction experiments for each baseline dataset. This approach ensured that we leveraged the most effective LLM-generated data for each dataset, rather than simply increasing synthetic data volume without considering quality. The average F1-scores obtained from these experiments are reported in Table 7.

For SMM, Diffusion-TS (−8.11%) and the Auto configuration (−15.41%) degraded performance, indicating poor generalization of these synthetic datasets. The text-to-motion ensemble (T2M+ParCo+SATO) remained relatively stable with a minor decline (−1.35%), while the FS text-to-text models (Gemini+GPT4+GPT4o) showed a small improvement (+0.81%), surpassing the baseline. For KFall, the Auto configuration achieved the highest gain (+15.94%), followed by Diffusion-TS (+9.77%) and the text-to-motion ensemble (+8.74%). FS text-to-text models contributed a moderate gain of +5.91%. For UMAFall, Auto-synthetic data again produced the highest improvement (+27.30%), followed closely by the text-to-motion ensemble (+25.46%), Diffusion-TS (+20.30%), and FS text-to-text models (+18.45%). For SisFall, improvements were modest. FS text-to-text models led with the highest gain (+4.10%), followed by Diffusion-TS (+2.19%), text-to-motion models (+1.64%), and Auto-synthetic data (+0.82%).

**Findings:** The results indicate that increasing synthetic data can improve model performance, but its effectiveness depends on the dataset and the type of synthetic data used. For SMM, text-to-text synthetic data led to a slight improvement, whereas an excessive amount of synthetic motion data did not provide significant benefits. In KFall and UMAFall, increasing synthetic data substantially boosted accuracy, with Auto-generated text-based falls and text-to-motion models yielding the highest gains. However, for SisFall, the performance gains were modest, suggesting that higher proportions of synthetic data may not always benefit models trained on high-frequency datasets. These findings confirm that LLM-generated synthetic data quantity does influence model accuracy, but its impact varies across datasets.

## 7. Key Findings and Applicability of LLMs for Fall Detection

After conducting a comprehensive experimental analysis, this section highlights the key findings of our study by addressing the research questions posed in the introduction. To provide a structured overview of these findings, Table 8 summarizes the comparative performance, strengths, and limitations of each generative approach, across critical dimensions such as joint specificity, demographic sensitivity, alignment with real data, fall detection performance, and scalability.

1. **Can LLMs generate accelerometer data specific to gender, age, and joint placement?**
  Our results indicate that text-to-motion models (T2M, SATO, and ParCo) effectively capture joint-specific variations but fail to differentiate demographic attributes such as gender and age. On the other hand, text-to-text models (GPT4o, GPT4, and Gemini) struggled to introduce both joint and demographic variations, producing only minor differences in joint-specific prompts, making them less effective for applications requiring age- or gender-specific biomechanical variations.
2. **Do LLM-generated data align more closely with real data than diffusion-based methods?**
  When evaluating alignment with real data, Diffusion-TS consistently outperformed all LLM-generated data, achieving the lowest JSD and highest Coverage scores across all datasets. Among LLM-based methods, text-to-motion models (T2M, SATO, and ParCo) demonstrated better alignment than text-to-text models, capturing more realistic motion distributions. However, text-to-text models exhibited high variance and spikiness, particularly in datasets with higher sampling rates such as KFall (100 Hz) and SisFall (200 Hz). This suggests that LLMs lose specificity as the sampling rate increases, leading to poor alignment. Although few-shot prompting slightly improved data realism, it also introduced noise and instability, particularly in high-frequency datasets, further limiting the reliability of LLM-generated synthetic data.
3. **Do LLM-generated data improve fall detection model performance?**
  The impact of LLM-generated data on fall detection performance varied by dataset characteristics. In the SMM dataset, performance declined across all synthetic methods, with T2M causing the least drop (−4.05%). This reduction was largely due to two factors: (1) the use of a left wrist sensor, which captures subtle, low-amplitude movements that provide weak fall indicators, and (2) temporal mismatch, i.e., SMM data includes only the impact phase, while synthetic data modeled full transitions, leading to poor alignment. In contrast, UMAFall showed the greatest performance gain, with GPT4-FS improving the F1-score by +56.83%. This improvement is likely due to the low sampling rate (20 Hz) of the UMAFALL dataset and limited activity complexity, which made it easier for synthetic data to complement the original distribution. KFall and SisFall experienced moderate improvements, although high-frequency sampling in SisFall (200 Hz) made it more sensitive to noise introduced by FS prompting. Specifically, GPT4o-FS reduced performance in SisFall by −7.10%. Overall, text-to-motion models consistently outperformed text-to-text models in maintaining alignment with baseline data. While Diffusion-TS offered the most stable results across all datasets, it showed limited performance gain, suggesting that its well-formed outputs may lack critical fall-specific variability required for generalization.

**Applicability of LLMs for Fall Detection**

Despite the improvements in F1-score, the highest gain (56.83% in UMAFall) remains below 90%, which is insufficient for real-world deployment. When implemented in wearable devices, quantization further reduces accuracy, making these marginal improvements negligible. Moreover, prompt engineering is labor-intensive and time-consuming, requiring multiple iterations to generate usable data. Even auto-generated prompts lack consistency, and dependency on chatbot APIs introduces variability, making real-time data generation impractical. These limitations, along with instability in high-frequency data, highlight the scalability challenges of LLMs. Nevertheless, LLMs provide valuable insights into joint-specific generation and serve as a flexible tool for scenario-driven data augmentation.

A potential alternative is to develop a foundation model for time-series synthesis, trained on a vast corpus of multimodal sensor data (e.g., accelerometer, gyroscope, and magnetometer data), capturing diverse activities and sensor configurations. If properly conditioned, such a model could generate fall-specific data aligned with demographic and sensor-specific properties. However, this approach is constrained by data scarcity and the difficulty of generating fall data that precisely mimics biomechanical transitions rather than repurposed generic movements.

In contrast, fine-tuning text-to-motion models (T2M, ParCo, and SATO) offers a more practical and targeted solution for fall-specific data generation. These models generate full-body joint trajectories from which accelerometer signals can be extracted at specific joints. This approach enables control over sensor placement and better biomechanical accuracy. However, we acknowledge that fine-tuning these models introduces additional computational costs and resource demands, which must be considered in real-world deployments.

Given the challenges in collecting diverse demographic-specific fall data, fine-tuned text-to-motion models remain the most viable method at present. Future research should address automation of prompt optimization, sensor-specific alignment, and lightweight deployment strategies to bridge the gap between synthetic data generation and scalable fall detection systems. These steps are critical to translating current findings into deployable solutions for real-world use.

## 8. Conclusions and Future Work

The difficulty of collecting real fall data, particularly from elderly individuals, poses a significant challenge for developing effective fall detection systems. To address this, we explore the use of Large Language Models (LLMs) for generating synthetic accelerometer data that can augment real datasets and potentially enhance model performance. This study is guided by three core research questions: (1) Can LLMs generate accelerometer data specific to joint placement or demographic features? (2) Do LLM-generated data better align with real data than diffusion-based models? (3) Do LLM-generated data improve fall detection performance?

To investigate these questions, we used two categories of LLMs, text-to-motion models (T2M-GPT, SATO, and ParCo) and text-to-text models (GPT4o, GPT4, and Gemini), and compared them with Diffusion-TS. Text-to-motion models were used to synthesize joint-specific signals from generated motions, while text-to-text models were prompted to directly output fall-related accelerometer sequences. The generated data were used to augment four baseline fall datasets (UMAFall, KFall, SisFall, and SMM), and their impact was evaluated using an LSTM-based fall detection model and F1-score as the performance metric. Our findings show that dataset characteristics such as sampling rate, sensor placement, and motion complexity critically influence the effectiveness of synthetic data. Text-to-motion models generally outperformed text-to-text models, while Diffusion-TS provided stable but less impactful results. Ablation studies further revealed that prompt design, sensor modality, and the amount of LLM-generated synthetic data significantly affect its usefulness for fall detection.

To overcome the limitations of LLMs identified in this study, future work will focus on the following: (1) fine-tuning text-to-motion models on real fall data to improve biomechanical specificity and realism, (2) developing automated, self-optimizing prompt generation pipelines to reduce manual effort and improve consistency, (3) enhancing generation fidelity for high-frequency signals by incorporating sampling-aware constraints, and (4) building lightweight generative models that can be fine-tuned and executed on commodity hardware, such as smartphones and wearables, eliminating reliance on cloud-based APIs and enabling real-time, private, and scalable synthetic data generation. This work provides the first comparative analysis of LLM-based synthetic fall data generation and its impact on fall detection. By identifying key limitations and actionable directions, it lays the groundwork for future advancements toward reliable, scalable, and demographically inclusive fall detection systems.

## Figures and Tables

**Table 1:** Comparison of synthetic data generation approaches for HAR and fall detection. A checkmark (✓) indicates the method provides the listed capability, while a cross (✗) indicates its absence or limitation.

Method	Sensor Placement Control	Demographic Specificity	Temporal Dynamics	Limitations
GANs (WGAN, CTGAN, TGAN)	✗	✗	Moderate	Mode collapse, tuning instability
Probabilistic Models (SDV-PAR)	✗	✗	Low–Moderate	Poor realism, limited expressiveness
Pose Estimation from Video	✓ (indirect)	✗	Moderate	Sensitive to occlusion, video quality
3D Simulation (ElderSim, I3D)	✓	✗	High	High compute cost, low diversity
Diffusion Models (M2D2M, MOTE)	✓	✗	High	Requires large data, lacks fine control
Digital Twins (OpenSim)	✓	✓ (potential)	Very High	Needs calibration, poor generalizability
LLMs (text-to-motion)	✓	✗	High	No demographic control, fine-tuning cost

**Table 2:** Summary of four baseline datasets considering the number of participants, their ages and genders (M: Males, and F: Females), number of falls and ADLs, sensors used, and sampling rate.

Dataset	Participants	Age (Years)	Gender (M/F)	Falls	ADLs	Sensors Used	Sampling Rate (Hz)
SMM	42 (16 young, 26 old)	23 (young), 65.5 (old)	11M, 5F (young); 12M, 14F (old)	5 types	9 types	Huawei Smartwatch (left wrist), Nexus Smartphone (right hip)	32
KFall	32 (all young)	24.9 (young)	32M, 0F	15 types	21 types	LPMS-B2 (lower back)	100
UMAFall	17 (all young)	19–28 (young)	10M, 7F	3 types	8 types	SimpleLink SensorTag (waist, right wrist)	20
SisFall	38 (23 young, 15 old)	19–30 (young), 60–75 (old)	11M, 12F (young); 8Males, 7F (old)	15 types	19 types	ADXL345 (waist)	200

**Table 3:** List of prompts used for synthetic data generation.

Index	Prompts
1	An elderly person is falling down.
2	An elderly person sits, becomes unconscious, then falls on his left, and lies on the floor.
3	An elderly person walks, becomes unconscious, then falls on his head, and lies on the floor.
4	An elderly person walks, then he falls down on his face, and stays on the floor.
5	An elderly person falls down on his right side, and lies on the ground.
6	A person stands still, wobbles, leans right suddenly, then falls down, and tucks in tight on the floor.
7	A person walks, slips suddenly, and falls on his back on the floor.
8	A person climbs a ladder, loses grip on one rung, and falls sideways.
9	A person walks on gravel, twists their ankle, and falls forward onto their hands and knees down to the floor.
10	A person runs, then slips and falls flat on the floor.
11	A person walks, suddenly trips, loses his balance, and ends up lying on the floor.
12	A person runs, slips, and FALLS FLAT on the floor.
13	A person walks, suddenly trips, loses his balance, and falls down on the floor, unable to get up.
14	A person walks, suddenly trips, loses his balance, and falls down and then ends up lying on the floor.
15	A person missed a chair and fell on the floor on his head.
16	A person falls asleep, falls down on the floor on his head.
17	A person is standing up, and falls down on the floor on his head.
18	A person falls down on the floor on his head.
19	A person walks, falls asleep, and then falls down on the floor on his head.
20	A child rides a bike too fast, loses control, and tumbles onto the grass with a scraped knee.
21	A person is stepping up, and falls down on the floor on his head.
22	A person walks, and then falls down on his head on the floor.
23	A person stands up from a chair, and falls down on the floor on his head.
24	A person bends to pick an object, and falls down on the floor on his head.
25	A person climbs stairs and falls down on the floor on his head.
26	A person kneels down to pick an object, slips, and falls on his head on the floor.
27	A person missed a chair and fell on the floor on his left.
28	A person missed a chair and fell on the floor on his back.
29	A person bends to the left, picks up something, becomes unconscious, and falls down onthe floor on his head.
30	A person turns right, and then falls down on the floor on his head.
31	A person walks forward, becomes unconscious, and falls down on the floor on his head.
32	A person is walking. They suddenly trip, lose their balance, and end up lying on the floor.
33	A person walking suddenly trips, loses their balance, and ends up lying on the floor, unable to get up.
34	A person walking suddenly trips, loses their balance, and ends up lying on the floor.
35	A person is standing upright, then suddenly trips and ends up lying in the fetal position.
36	A person stands up. Then they descend to the floor, lies sprawled out.
37	A person attempts to get out of their chair, but ends up hitting their head on the floor and lying sprawled out.
38	A person is standing upright. They do a half turn before they lose their balance and descend to the floor. They lie with their legs pulled up to their chest.
39	A person is taking confident strides, until they trip hitting their head as they lie on the ground sprawled out.
40	A person walks forward and trips ending on the floor.
41	A person walks backwards and trips and falls ending on the floor.
42	A person walks on a wet floor, slips, then falls and lands on their side.
43	An elderly person stumbles while stepping off a curb, falls, and lands on their hip.
44	A person reaches for an object on a high shelf, loses balance, and falls backward on the floor.
45	A person runs down a hill, loses control, and rolls to a stop on the ground.
46	A person stands on a chair to change a light bulb, the chair tips, and they fall to the floor.
47	A person trips over his own feet, falls on the ground, and ends up lying there.
48	A person rides a scooter, hits a bump, and falls, landing on their knee.
49	A person leans too far back in a chair, and he falls backward on the floor.
50	A person walks downstairs, missteps, and falls down on the floor on his back.

**Table 4:** Performance comparison of various synthetic data generation methods for fall detection, showing percentage increase (+) or decrease (−) in the average F1-score of the LSTM model relative to the baseline. Results are reported across 5-fold cross-validation. Bold values indicate the best-performing synthetic data generation method for each dataset.

Dataset	Baseline	Diffusion-TS	Text-to-Motion LLMs			Text-to-Text LLMs					
			T2M	ParCo	SATO	Gemini-Flash-8B		GPT4 (Copilot)		GPT4o (ChatGPT)	
						Zeroshot	Fewshot	Zeroshot	Fewshot	Zeroshot	Fewshot
SMM	0.740	0.680 (−8.11%)	**0.710 (−4.05%)**	0.680 (−8.11%)	0.648 (−12.43%)	0.636 (−14.05%)	0.662 (−10.54%)	0.578 (−21.89%)	0.626 (−15.41%)	0.588 (−20.54%)	0.620 (−16.22%)
KFall	0.778	0.854 (+9.76%)	**0.902 (+15.94%)**	0.870 (+11.84%)	**0.902 (+15.94%)**	0.794 (+2.06%)	0.848 (+8.99%)	0.878 (+12.87%)	0.822 (+5.65%)	0.898 (+15.43%)	0.892 (+14.65%)
UMAFall	0.542	0.652 (+20.30%)	0.630 (+16.23%)	0.698 (+28.78%)	0.712 (+31.18%)	0.575 (+6.09%)	0.702 (+29.52%)	0.630 (+16.23%)	**0.850 (+56.83%)**	0.580 (+7.01%)	0.756 (+39.48%)
SisFall	0.732	0.748 (+2.19%)	0.742 (+1.37%)	0.758 (+3.55%)	**0.784 (+7.10%)**	0.752 (+2.73%)	0.750 (+2.46%)	0.772 (+5.46%)	0.776 (+6.01%)	0.782 (+6.83%)	0.680 (−7.10%)

**Table 5:** Impact of baseline dataset characteristics on LSTM-based fall detection: Average F1-scores (+/−%) across 5-fold cross-validation. Bold values represent the highest gains for each dataset.

Dataset	Baseline	Diffusion-TS	Text-to-Motion LLMs			Text-to-Text LLMs					
			T2M	ParCo	SATO	Gemini-Flash-8B		Copilot		GPT4o	
						Zeroshot	Fewshot	Zeroshot	Fewshot	Zeroshot	Fewshot
SMM (right hip)	0.8075	0.778 (−3.65%)	0.812 (+0.56%)	**0.856 (+6.01%)**	0.746 (−7.62%)	0.762 (−5.63%)	0.682 (−15.54%)	0.806 (−0.19%)	0.754 (−6.63%)	0.772 (−4.40%)	0.754 (−6.63%)
UMAFall (waist)	0.744	0.922 (+23.92%)	0.854 (+14.78%)	0.850 (+14.25%)	0.892 (+19.89%)	**0.946 (+27.15%)**	0.710 (−4.57%)	0.888 (+19.35%)	**0.946 (+27.15%)**	0.888 (+19.35%)	0.702 (−5.65%)

**Table 6:** Comparison of auto-generated and manually designed prompts: Impact on synthetic data quality and fall detection performance. Bold values represent the highest gains for each dataset.

Dataset	Baseline	GPT4o		Copilot		Gemini-Flash-8B	
		Zeroshot	Auto	Zeroshot	Auto	Zeroshot	Auto
SMM (left wrist)	0.740	0.588 (−20.54%)	**0.724 (−2.16%)**	0.578 (−21.89%)	0.714 (−3.51%)	0.636 (−14.05%)	0.626 (−15.41%)
KFall (waist)	0.778	**0.898 (+15.42%)**	0.892 (+14.65%)	0.878 (+12.85%)	0.874 (+12.34%)	0.794 (+2.06%)	0.850 (+9.25%)
UMAFall (waist)	0.542	0.580 (+7.01%)	**0.840 (+54.98%)**	0.630 (+16.24%)	0.660 (+21.77%)	0.575 (+6.09%)	0.723 (+33.39%)
SisFall (waist)	0.732	**0.782 (+6.83%)**	0.768 (+4.92%)	0.772 (+5.46%)	0.774 (+5.74%)	0.752 (+2.73%)	0.772 (+5.46%)

**Table 7:** Impact of increasing synthetic data proportion up to 80% on fall detection performance. Bold values represent the highest gains for each dataset.

Dataset	Baseline	Diffusion-TS	Text-to-Motion Models (T2M+ParCo+SATO)	Text-to-Text Models (Gemini+GPT4+GPT4o)	Auto (Gemini+GPT4+GPT4o)
SMM (left wrist)	0.740	0.680 (−8.11%)	0.730 (−1.35%)	**0.746 (+0.81%)**	0.626 (−15.41%)
KFall (waist)	0.778	0.854 (+9.77%)	0.846 (+8.74%)	0.824 (+5.91%)	**0.902 (+15.94%)**
UMAFall (right wrist)	0.542	0.652 (+20.30%)	0.680 (+25.46%)	0.642 (+18.45%)	**0.690 (+27.30%)**
SisFall (waist)	0.732	0.748 (+2.19%)	0.744 (+1.64%)	**0.762 (+4.10%)**	0.738 (+0.82%)

**Table 8:** Summary of model capabilities and limitations for synthetic fall data generation. The entry “N/A” indicates the aspect is not applicable.

Aspect	Text-to-Text LLMs (GPT4, GPT4o, Gemini)	Text-to-Motion Models (T2M, SATO, ParCo)	Diffusion-TS	Remarks
Joint-specific motion	Limited	Strong	N/A	T2M models capture body-part motion more realistically
Demographic sensitivity (age/gender)	Weak	Weak	N/A	None of the models showed demographic variation
Alignment with real data (JSD, Coverage)	Moderate to poor, unstable in high frequency	Moderate	Strong	Diffusion-TS maintains stability across datasets
Fall detection performance (F1-score)	Dataset-dependent; best on UMAFall	Consistent gains across datasets	Stable but not always highest	T2M often yields highest gains
Sampling rate robustness	Poor at high frequency (≥100 Hz)	Moderate	Strong	LLMs degrade as sampling rate increases
Prompt scalability	Manual or Auto; labor-intensive	Manual; labor-intensive	N/A	Prompt engineering is a major bottleneck for LLMs
Computational cost	Low (inference only)	High (requires fine-tuning)	Moderate (training required)	T2M fine-tuning is costly but effective
Deployability on wearables	Limited (due to quantization, noise)	Limited (requires model compression)	More practical	Diffusion-TS offers better stability

## Data Availability

The synthetic IMU fall dataset generated in this study is publicly available at: https://github.com/txst-cs-smartfall/LLM-based-synthetic-fall-data-generation (accessed on 8 July 2025).
